# Early detection of COPD patients’ symptoms with personal environmental sensors: a remote sensing framework using probabilistic latent component analysis with linear dynamic systems

**DOI:** 10.1007/s00521-023-08554-5

**Published:** 2023-04-30

**Authors:** Şefki Kolozali, Lia Chatzidiakou, Roderic Jones, Jennifer K. Quint, Frank Kelly, Benjamin Barratt

**Affiliations:** 1 School of Computer Science and Electronic Engineering, University of Essex, Colchester, UK; 2 Department of Chemistry, University of Cambridge, Cambridge, UK; 3 Faculty of Medicine, National Heart and Lung Institute, Imperial College London, London, UK; 4 Faculty of Medicine, School of Public Health, Imperial College London, London, UK

**Keywords:** Internet of things (IoT) in Healthcare, Remote monitoring systems, Personal air pollution exposure, Chronic obstructive pulmonary disease (COPD), Probabilistic latent models

## Abstract

In this study, we present a cohort study involving 106 COPD patients using portable environmental sensor nodes with attached air pollution sensors and activity-related sensors, as well as daily symptom records and peak flow measurements to monitor patients’ activity and personal exposure to air pollution. This is the first study which attempts to predict COPD symptoms based on personal air pollution exposure. We developed a system that can detect COPD patients’ symptoms one day in advance of symptoms appearing. We proposed using the Probabilistic Latent Component Analysis (PLCA) model based on 3-dimensional and 4-dimensional spectral dictionary tensors for personalised and population monitoring, respectively. The model is combined with Linear Dynamic Systems (LDS) to track the patients’ symptoms. We compared the performance of PLCA and PLCA-LDS models against Random Forest models in the identification of COPD patients’ symptoms, since tree-based classifiers were used for remote monitoring of COPD patients in the literature. We found that there was a significant difference between the classifiers, symptoms and the personalised versus population factors. Our results show that the proposed PLCA-LDS-3D model outperformed the PLCA and the RF models between 4 and 20% on average. When we used only air pollutants as input, the PLCA-LDS-3D forecasting results in personalised and population models were 48.67 and 36.33% accuracy for worsening of lung capacity and 38.67 and 19% accuracy for exacerbation of COPD patients’ symptoms, respectively. We have shown that indicators of the quality of an individual’s environment, specifically air pollutants, are as good predictors of the worsening of respiratory symptoms in COPD patients as a direct measurement.

## Introduction

Obtaining comprehensive information about patients’ daily symptoms and exposure to acute risk factors can aid doctors in the accuracy of the diagnosis process as well as in the rehabilitation of patients. Moreover, it can assist patients by suggesting alterations to their behaviour to prevent worsening of their symptoms. Having greater evidence-based information can also help policymakers to make better decisions to minimise citizens’ risk, improving both their health and quality of life. According to the medical literature, daily self-reported symptoms correlate with patients’ deterioration. Provision of a personal digital health system that encapsulates the recording of daily symptoms, personal environmental exposure and activities of patients’ could help provide a solution for effective self-monitoring of symptoms and vital signs. Moreover, it could prevent or decrease hospital admissions caused by severe worsening of symptoms (“exacerbations”) using such systems to notify patients as early as possible. Such a system needs to learn and adapt to each patient’s symptom responses and dynamic environment, while taking into account the overall pattern.

While there are conventional physiological factors that can be used in the prediction systems [1–3], such as body weight, pulse rate, SpO2, there still remains a need for investigation to understand the effects of environmental factors. Moreover, factors such as bodyweight can be chronic predictors, but not acute day to day predictors, which is what we aim to demonstrate in this study. By utilising environmental factors in the prediction, we aim to design behavioural interventions to reduce risk of worsening of symptoms and/or exacerbation. These interventions would be in addition to any clinical interventions. The two approaches are complementary, not duplicative.

This is the first study which attempts to predict COPD symptoms based on personal air pollution exposure observed by using portable environmental sensor nodes with attached air pollution sensors. In this study we further extend our preliminary work in [4] by conducting numerous new experiments that take into account the following factors: (i) five different classification models that includes two new models that can take into account the season of the year; (ii) population versus personalised models in order to explore the effect of number of participants on our models; (iii) with and without personal coverage threshold to investigate the performance in symptom identification when participants carry our sensors and when they leave it at home running; (iv) three different sets of sensory input variation to quantify the effect of all sensory input, air pollutants and peak flow separately; (v) two different feature sets to explore the effect of rich spectral feature set versus simple statistical features.

## Background

There have been several remote monitoring studies in the detection of exacerbations of COPD patients’ symptoms and various prediction models and data modalities used in these studies [1–3, 5–7]. A Bayesian Network Model was applied to self-reported symptoms and peak flow measurements in order to predict the exacerbations of COPD patients in [5]. A Linear Discriminant Classification technique applied to systolic and diastolic blood pressure, pulse, saturation in [1]. In contrast to our study, the authors removed the recovery period (i.e. transient of symptom) from their data set in [1]. In [2], a multi-level logistic regression model was used to predict exacerbations from oxygen saturation, pulse rate and peak flow measurements. These studies did not address the forecasting of exacerbations of COPD patients.

In [6], the researchers applied a Probabilistic Neural Network on daily questionnaire data to predict the exacerbations of COPD patients’ symptoms. There is not adequate information regarding the training and testing methodology of this study nor a detailed analysis of the predictions made by this model. Moreover, although the authors argue that it is an early prediction system, there is little evidence that this system is a forecasting system. In another study [3], a regression tree and relatively rich physiological parameters, such as respiratory and heart rate, body weight and temperature, and peak flow measurements, were used to forecast exacerbations one day in advance. They found that the most predictive power was obtained by using bodyweight, SpO2 and peak flow measurements. In [7], a k-means clustering approach was used for forecasting the exacerbations based on questionnaire data. However, the authors did not include the recovery period in their analysis. They found that they can forecast the onset of exacerbations an average of 4 to 6 days in advance. Nonetheless, these studies fell short in using dynamic systems in the forecasting process and did not use any sliding windows to process data continuously. It is possible to find an extensive review of recent studies about remote monitoring of COPD patients in [8].

While most of the studies mentioned above managed to obtain reasonably good results in their predictions, the majority did not focus on forecasting exacerbations, nor did they incorporate personal air pollution exposure measurements. Therefore, our study is the first of its kind to attempt forecasting symptoms and exacerbations as well as all three temporal states of symptoms (i.e. onsets, transients, offsets) directly from high-resolution personal air pollution exposure measurements and peak flow measurements. Having such systems could provide GPs with evidence-based information and could be used for behavioural interventions to reduce risk of worsening of symptoms and/or exacerbations.

**Fig. 1:**
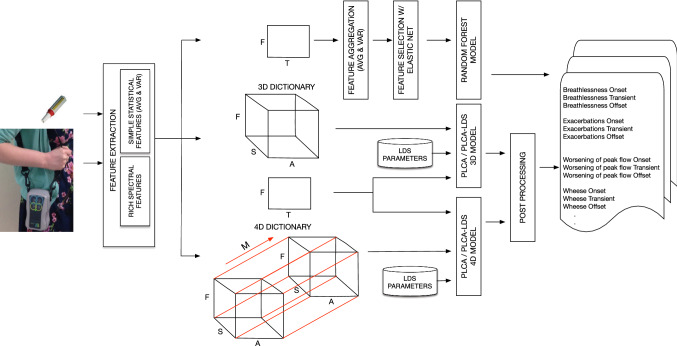
The proposed framework for the prediction of patients’ COPD symptoms. The Personal Air Monitor (PAM) being carried on the bottom left and peak flow meter on the top left. Corresponding meanings of the abbreviations are as follows: *T*: Time (daily); *S*: Symptoms; *A*: Temporal state of a symptom (i.e. Onset, Transient, Offset); *F*: Spectral features extracted from sensory data; *M*: Seasons. The window size is 8 days and step size is 1 day

## Methodology

### Data collection methodology

Patients were approached by GPs through the Clinical Practice Research Datalink (CPRD), an anonymised general practice records database containing ongoing primary care medical data [9]. Participants were invited to a clinic and provided with a PAM (see Fig. 1). They were instructed to keep the monitor at home and take it out with them for a minimum of once a week for up to 6 months. At the beginning of the monitoring phase, the participants filled a questionnaire to provide information regarding their lifestyle, and residence characteristics, including type of cooker used in the home (e.g. wood burning stove, gas, or electric) and car ownership. Moreover, spirometry readings were collected at the initial appointment and subsequent follow-up visits as appropriate. During the monitoring period, participants completed daily diary cards of their symptoms, any changes in their medications and treatment, and sleep disturbance, and to measure and record their peak expiratory flow using a peak flow meter by taking the average of three consequent peak flow measurements. We used a spirometer device in the evaluation of the COPD patients at the hospital. A replacement was recruited if at any stage the wearing compliance of the PAM was low, or the participant chosen to withdraw. Throughout the monitoring period, participants received phone calls from the research assistant to check how they were coping with the study. Six weeks into the monitoring period, participants were suggested to visit a clinic with the research assistant in order to discuss any problems with the PAMs or diary cards. At the end of the monitoring period, participants were invited to a final appointment to return the PAMs and completed diary cards. More detailed information about the data collection methodology can be found in [10] and about the instruments used in the study can be found in [11–13].

### Motivation and system overview

Figure 1 illustrates the proposed framework. The overall aim of the proposed framework is the creation of a system for the detection of worsening of COPD patients’ symptoms which can also support multivariate tracking of symptoms over time. A comprehensive comparison has been made between Bayesian Tensor Factorisation and various feature selection techniques and traditional classifiers (i.e. RFs, Lasso, SVM) in [14]. While the results showed that Tensor Factorisation techniques have outperformed the traditional techniques when the dimensionality of datasets increased, the obtained eigen tensors need to be fed into a classifier to carry out a classification task as in [15]. The shortcoming of these studies is either they cannot deal with multi-label classification or time series prediction problems. Instead, we used a PLCA method [16] that can predict multiple sets of outcomes in high dimensional tensor format at the same time. The tensor and matrix factorisation techniques do not model the transition of time-axis and produce continuous output. Therefore, it is possible to get fragmented output rather than a smooth predictive outcome. As a solution, we applied a hybrid approach, that is proposed in [4, 17]. The probabilistic approach combines the PLCA model with Markov chain of latent variables or State Space Models, where each observation conditioned on the state of the corresponding latent variable. We compared our model against Random Forest (RF) classifier. It is a multi-class and multi-label classifier and proven to be a powerful pattern recognition technique. Indeed, RF was recently shown the best performance among 179 classifiers arising from 17 families (e.g. Bayesian, Support Vector Machines, Neural Networks, boosting, bagging, nearest neighbours) on 121 data sets from University of California, Irvine (UCI) Machine Learning Repository^1^ in an extensive study [18]. Although it was not an RF model, Tree Classifiers were also used in a recent study on remote monitoring of COPD patients [3].

We developed two different PLCA models: (i) personalised PLCA models per participant (i.e. a 3rd order dictionary tensor: symptom type, temporal state of the symptoms, and frequency) and (ii) a population PLCA model (4th order dictionary tensor: patients, symptom type, temporal state of the symptoms, and frequency). The system takes multi-channel sensory observations as input. The specific observations include Nitric Oxide (NO), Carbon Monoxide (CO), Particulate Matter $<1$ $\upmu$m in diameter (PM1), Particulate Matter < 2.5 $\upmu$m in diameter (PM2.5), Particulate Matter $<10$ $\upmu$m in diameter (PM10), relative humidity (RH), background noise, triaxial accelerometer, temperature, spatial coordinates (GPS). Daily peak flow measurements of each participant were also used in our experiments as an input and as a binarised output obtained based on each participant’s median peak flow measurements. The model uses a pre-extracted dictionary of spectral templates in the form of tensors. Non-negative Matrix Factorisation (NMF) approach used in the creation of the dictionary templates. Symptom tracking using LDS can take place within the PLCA inference or can take place as a post-processing step. We used the LDS within the PLCA model. The model output is finally converted into a list of symptoms identified along with the temporal states, such as onset, transient, and offset. Then, a thresholding approach is applied to the output, where we compute sets of metrics, F-measure, for all possible permutations and use the best threshold values for each symptom in the testing phase.

### Preprocessing

The lags determines how often an incident may occur. In our experiments, we computed lags based on peak flow measurements of patients in order to determine the window size. We individually calculated autocorrelations and lags of patients and then calculated the average and median of the outputs to determine the window size for entire cohort. We used the small lag as window size (i.e. 8 days) both for environmental and health data streams. We used Discrete Wavelet Transform (DWT), particularly Daubechies wavelets to decompose the environmental data into lower resolution components, and extracted magnitude, acute and cumulative spectral and statistical features, such as Spectral Flux, Spectral Centroid, Spectral Energy, Average, Median, Kurtosis, Variance, Skewness using a sliding window in the feature extraction phase with a window size of 8 days.

### Probabilistic latent component analysis (PLCA)

The applied PLCA models take multi-channel sensor readings, $V_{f,t}$ and approximates it as a bivariate probability distribution over time and frequency, *P*(*f*, *t*). This quantity then is factored into a frame probability *P*(*t*), which is computed directly from the observed data, and a conditional distribution over frequency $P(f \mid t)$. The frames are treated as repeated draws from an underlying random process characterised by $P(f\mid t)$. It can model this distribution with a mixture of latent factors as follows:

1 $$\begin{aligned} P(f, t)\,=\, & {} P(t)P(f\mid t) \nonumber \\\,= \,& {} P(t) \sum _{z} P(f\mid z)(P(z\mid t) \end{aligned}$$

where *z* corresponds to the component index, *P*(*t*) is the *l*1 norm for the *t*-th spectral features frame (a known quantity), $P(f\mid z)$ is the spectral template that corresponds to the *z*-th component, and $P(z\mid t)$ is the activation of the *z*-th component over *t*. It is effectively same as NMF since there is only a single latent variable *z* in Eq. (1). However, it has an advantage of probabilistic interpretation, which enables us to introduce additional parameters and constraints.

#### PLCA 3D

Suppose now that we wish to model a mixture of *S* symptoms, where each source has *A* possible temporal states. The PLCA allows us to extends the model described in Eq. (1) to accommodate these parameters as follows:

2 $$\begin{aligned} P(f\mid t)= P(t) \sum _{s,a} P(f\mid s,a)P(s\mid t) P(a\mid s,t) \end{aligned}$$

The model decomposes the approximated spectral features *P*(*f*, *t*) into a dictionary of spectral templates per symptom *s*, and temporal state of symptom *a*, as well as probability distributions for symptom activations. We developed two different models for our experiments, namely, PLCA-3D and PLCA-4D models. The PLCA-3D model is used in both personalised and population level symptom detection. It is formulated as in Eq. (2). The PLCA-4D model is only used in the population study, where we extended the previous model by taking into account seasonality of symptom, *m*, in order to see the effect of seasonal change on the symptom detection. It is formulated as in Eq. (3):

3 $$\begin{aligned}{} & {} P(f\mid t) \nonumber \\{} & {} \quad = P(t) \sum _{m,s,a} P(f\mid m,s,a) P(s\mid t) P(a\mid s,t)P(m\mid s,t) \end{aligned}$$

where *s* $\in$ $\{1,...,S\}$ denotes the symptom class, *a* $\in$ $\{1,...,A\}$ denotes the temporal state of symptom, and *m* $\in$ $\{1,...,M\}$ denotes the seasonality of symptom. *P*(*t*) is defined as $\sum _{f}V_{f,t}$, which is a known quantity. It corresponds to the sum of all spectral features for each time frame *t*. Each *t* corresponds to spectral features calculated for the last 8 days. For the PLCA-3D, by applying NMF on the extracted features, we obtained a 3-dimensional tensor dictionary $P(f\mid a,s)$ that contains the spectral features for each patient’s symptom *s*, temporal state *a*. $P(s\mid t)$ is the time-varying symptom activation. $P(a\mid s,t)$ represents the activation of temporal state of each symptom, *s*, across time *t*. Similarly, for the PLCA-4D, we prepared a 4-dimensional tensor dictionary $P(f\mid m,s,a)$ that represents the spectral features, *f*, symptoms, *s*, temporal states of symptoms, *a*, and additionally, the seasonality of symptoms, *m*. $P(m\mid s,t)$ represents the seasonality activation per symptom *s*, across time *t*.

To be regarded as probabilities, spectral features $P(f\mid s,a)$ are normalised with respect to *f* as to sum to one. Moreover, $P(s\mid t)$, $P(a\mid s,t)$, and $P(m\mid s,t)$ are similarly normalised with respect to *s*, *a*, and *m*, respectively. On the contrary, $P(f\mid t)$ and *P*(*t*) are not normalised since they carry information on the energy of the spectral features. Nonetheless, since *P*(*t*) and *P*(*f*, *t*) are cancelled out through the partition functions, this doesn’t affect inference. The unknown model parameters, $P(s\mid t)$, $P(a\mid s,t)$, and $P(m\mid s,t)$, were estimated using iterative update rules, Expectation-Maximisation (EM) algorithm. For the *E-step* of the PLCA-3D model, the following posterior is computed:

4 $$\begin{aligned} P(s,a\mid f,t) \,\,=\,\, \frac{P(f\mid s,a)P(s \mid t)P(a\mid s,t)}{\sum _{s,a}P(f\mid s,a)P(s\mid t)P(a\mid s,t)} \end{aligned}$$

For the *M-step* of PLCA-3D model, $P(s\mid t)$ and $P(a\mid s,t)$ are updated using the posterior of Eq. (4):

5 $$\begin{aligned} P(s\mid t)\,\,=\,\, & {} \frac{\sum _{a,f}P(s,a\mid f,t)V_{f,t}}{{\sum _{s,a,f}P(s,a\mid f,t)V_{f,t}}} \end{aligned}$$

6 $$\begin{aligned} P(a\mid s,t)\,\,=\,\, & {} \frac{\sum _{f}P(s,a\mid f,t)V_{f,t}}{\sum _{a,f}P(s,a,\mid f,t)V_{f,t}} \end{aligned}$$

#### PLCA 4D

In our population study, we used the PLCA-4D model, where we incorporated a seasonality variable, *m*, in our model to capture the seasonal change in our predictions. Specifically, it captures 4 different seasons (i.e. spring, summer, autumn, winter) in our model. Thus, we formulated our problem by using the following equations. For the *E-step* of the the PLCA-4D, the following posterior is computed:

7 $$\begin{aligned}{} & {} P(m,s,a\mid f,t) \nonumber \\{} & {} \quad =\frac{P(f\mid m,s,a)P(s\mid t)P(m\mid s,t)P(a\mid s,t)}{\sum _{m,s,a}P(f\mid m,s,a)P(s\mid t)P(m\mid s,t)P(a\mid s,t)} \end{aligned}$$

For the *M-step* of the PLCA-4D model, $P(s\mid t)$, $P(a\mid s,t)$, and $P(m\mid s,t)$ are updated using the posterior of Eq. (7):

8 $$\begin{aligned} P(s\mid t)\,\,=\,\, & {} \frac{\sum _{m,a,f}P(m,s,a\mid f,t)V_{f,t}}{{\sum _{m,s,a,f}P(m,s,a\mid f,t)V_{f,t}}} \end{aligned}$$

9 $$\begin{aligned} P(a \mid s,t),\,=\,\, & {} \frac{\sum _{m,f}P(m,s,a\mid f,t)V_{f,t}}{\sum _{m,a,f}P(m,s,a\mid f,t)V_{f,t}} \end{aligned}$$

10 $$\begin{aligned} P(m \mid s,t),\,=\,\, & {} \frac{\sum _{a,f}P(m,s,a \mid f,t)V_{f,t}}{\sum _{m,a,f}P(m,s,a\mid f,t)V_{f,t}} \end{aligned}$$

Once we obtained our models, we further apply constrains to deal with sparsity of certain unknown model parameters. Due to the fact that only a few symptom classes are expected to occur at a given time frame, we impose sparsity on the symptom detection $P(s\mid t)$. The sparsity constraints are applied by following a similar method to the one used in [19], by changing the update Eqs. (5), (8) as follows:

11 $$\begin{aligned} P(s\mid t)\,=\, & {} \frac{(\sum _{a,f}P(s,a\mid f,t)V_{f,t})^{\kappa }}{{\sum _{s}(\sum _{a,f}P(s,a\mid f,t)V_{f,t}})^{\kappa }} \end{aligned}$$

12 $$\begin{aligned} P(s\mid t)\,=\, & {} \frac{(\sum _{m,a,f}P(m,s,a\mid f,t)V_{f,t})^{\kappa }}{{\sum _{s}(\sum _{m,a,f}P(m,s,a\mid f,t)V_{f,t}})^{\kappa }} \end{aligned}$$

In order to lower the entropy in $P(s\mid t)$ and to promote sparsity, we set $\kappa$ $>1$ (i.e. typical values are between 1.1 and 1.5). We set $\kappa$ to 1.1. Due to the fact that $P(f\mid s,a)$ and $P(f\mid m,s,a)$ was pre-extracted and considered as fixed variable, we have not used any update rule for the symptom feature templates.

We initialised the unknown parameters $P(s\mid t)$, $P(a\mid s,t)$, $P(m\mid s,t)$ in the EM updates with random values between 0 and 1. We iterated Eqs. (5) and (6) for the PLCA-3D model, and Eqs. (8)–(10) for the PLCA-4D model until convergence. In our experiments, we found 40 iterations to be sufficient. For both of the PLCA models, the obtained output is a 2-dimensional non-binary representation of symptom activations over time, given by $P(s,t)=P(t)P(s\mid t)$ with dimensions of $S \times T$. Essentially, the output created by calculating the posterior probability of each symptom over all possible symptoms (i.e.  $P(s = 1\mid t), P(s = 2\mid t),...,P(s = S\mid t)$) weighted by energy of the spectral features. The PLCA model output *P*(*s*, *t*) contains the non-binary activation of overlapping symptoms *s* over time *t*. However the models of Eqs. (2) and (3) do not support any temporal constraints. Thus, they can lead to temporally fragmented output. Here, we used LDS to perform symptom tracking.

### Linear dynamic systems (LDS)

**Fig. 2:**
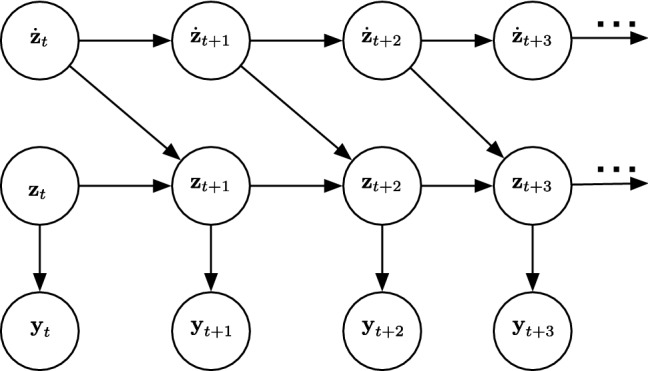
A graphical representation of a Linear Dynamic System with time derivatives for tracking. The hidden states, first derivative of hidden states, and observations, and time steps in tracking process are labeled as ${{\textbf {z}}}$, $\dot{{{\textbf {z}}}}$, ${{\textbf {y}}}$, *t*, respectively

LDS is a special case of State Space Models (SSM), where the latent and observed variables are multivariate Gaussian distributions, and their means are linear functions of their parent states. LDS estimates the state ${{\textbf {z}}} \in \mathfrak {R}^{n}$ of a discrete-time controlled process that is governed by the linear stochastic difference equation given below:

13 $$\begin{aligned} {{\textbf {z}}_{t+1}}={{\textbf {A}}}{{\textbf {z}}}_{t} + \epsilon _{t} \end{aligned}$$

14 $$\begin{aligned} {{\textbf {y}}}_{t+1}={{\textbf {H}}}{{\textbf {z}}}_{t+1} + \delta _{t} \end{aligned}$$

where ${{\textbf {z}}}_{t-1}$ is the hidden state, ${{\textbf {A}}}$ represents the transition model, and $\epsilon$ is the process noise, ${{\textbf {y}}}_{t}$ is the observation, ${{\textbf {H}}}$ is the observation model. In addition, the variable *t* represents the time step in the tracking process. The observations made are *m*-dimensional with a measurement ${{\textbf {y}}} \in \mathfrak {R}^{m}$. The random variables $\epsilon _{t}$ the process noise and $\delta _{t}$ represent the measurement noise. They are assumed to be independent of each other with normal probability distribution as given in the following equation:

15 $$\begin{aligned}&\epsilon _{t} \sim N (0,{{\textbf {Q}}}), \end{aligned}$$

16 $$\begin{aligned}&\delta _{t} \sim N (0,{{\textbf {R}}}) \end{aligned}$$

where ${{\textbf {Q}}}$ is the process noise covariance and ${{\textbf {R}}}$ is measurement noise covariance which change at each time step. Figure 2 shows a graphical illustration of an LDS system.

#### Learning LDS parameters

In our LDS model, we assume that symptom activation *P*(*s*, *t*) is a ‘noisy’ observation ${{\textbf {y}}}_{t}$ and latent states ${{\textbf {z}}}_{t}$ correspond to our desired output. Moreover, the latent variable space of our LDS model includes ‘velocity’ values $\dot{{{\textbf {z}}}}_{t}$ for each symptom class, signifying the difference in amplitude values in the symptom activation matrix *P*(*s*, *t*) across adjacent time frames. By initialising $P(s\mid t)$ in the EM updates with a binary mask that corresponds to the ground truth annotations, the resulting output only has nonzero activations in the time instants and classes corresponding to ground truth symptoms. Given a fully observed data, once can estimate transition states of the state space models, as it has been explained in [20]. Thus, given our fully observed data, we estimated the transition states, ${{\textbf {A}}}$ and ${{\textbf {H}}}$, by solving the least squares problem for ${{\textbf {z}}}_{t-1}\rightarrow {{\textbf {z}}}_{t}$ and ${{\textbf {z}}}_{t}\rightarrow {{\textbf {y}}}_{t}$.

17 $$\begin{aligned} J({{\textbf {A}}})= & {} \sum _{t} ({{\textbf {z}}}_{t} - {{\textbf {A}}}{{\textbf {z}}}_{t-1})^{2} \end{aligned}$$

18 $$\begin{aligned} J({{\textbf {H}}})= & {} \sum _{t} ({{\textbf {y}}}_{t} - {{\textbf {H}}}{{\textbf {z}}}_{t})^{2} \end{aligned}$$

Then, we assumed process and observation noise covariance matrices (i.e. ${{\textbf {Q}}}$ and ${{\textbf {R}}}$) to be diagonal in the form of ${{\textbf {Q}}}=\alpha$I and ${{\textbf {R}}}=\beta$I, which scaling parameters, $\alpha , \beta \in R$ estimated from training data. We set our $\alpha$ and $\beta$ to 0.2 and 0.1, respectively. Note that while ${{\textbf {A}}}$ represents transition states of LDS, *A* represents temporal states of symptoms in our equations.

#### LDS inference and postprocessing

In the inference phase, we estimate model posterior of the LDS model, which is represented as: $P({{\textbf {z}}}_{t}|{{\textbf {y}}}_{1:t}) = N({{\textbf {z}}}_{t}|\varvec{\mu }_{t},\varvec{\Sigma }_{t})$. We computed the posteriors by using the Kalman Filter equations given in [20–22]. Our probabilistic process is based on Gaussian distribution. Following the prediction step calculations of the stochastic difference equations given in Eqs. (13) and (14), once we initialised our covariance matrix, $E[({{\textbf {z}}}_{k}- \hat{{{\textbf {z}}}}_{k})({{\textbf {z}}}_{k}- \hat{{{\textbf {z}}}}_{k})^{T}]$, we calculated the prediction of the covariance matrix as follows:

19 $$\begin{aligned} \hat{\varvec{\Sigma }}_{t+1}={{\textbf {A}}\varvec{\Sigma }{} {\textbf {A}}}^{T}+ {{\textbf {Q}}} \end{aligned}$$

To update the estimate, we obtained the residuals, ${{\textbf {r}}}$, by the calculating the difference between our predicted observation and the actual observation as follow:

20 $$\begin{aligned}{} & {} {{\textbf {r}}}_{t+1}= {{\textbf {y}}}_{t+1} - \hat{{{\textbf {y}}}}_{t+1} \end{aligned}$$

21 $$\begin{aligned}{} & {} \hat{{{\textbf {y}}}}_{t+1} = {{\textbf {HA}}}\hat{{{\textbf {z}}}}_{t+1} \end{aligned}$$

In order to calculate the weight that needs to be placed on the error signal, we calculated the *Kalman gain* matrix, ${{{K}}}$, by following equation:

22 $$\begin{aligned} {{\textbf {K}}}_{t+1} = \hat{\varvec{\Sigma }}_{t+1} {{\textbf {H}}}^{T} + ({{\textbf {H}}} \hat{\varvec{\Sigma }}_{t+1} {{\textbf {H}}}^{T} + {{\textbf {R}}})^{-1} \end{aligned}$$

Subsequently, we minimised the Mean Squared Error (MSE) and updated the estimate for the state and covariance matrices:

23 $$\begin{aligned}{} & {} {{\textbf {z}}}_{t+1} = \hat{{{\textbf {z}}}}_{t+1} + ({{\textbf {K}}}_{t+1} {\textbf {r}}_{t+1}) \end{aligned}$$

24 $$\begin{aligned}{} & {} \varvec{\Sigma }_{t+1} = ({{\textbf {I}}} - {{\textbf {K}}}_{t+1} {{\textbf {H}}}) \hat{\varvec{\Sigma }}_{t+1} \end{aligned}$$

where ${{\textbf {I}}}$ is the identity matrix.The output of the symptom tracking process is the posterior mean $\varvec{\mu }_{t}$ (i.e. which is represented as ${{\textbf {z}}}_{t}$). After including the computed velocity into the hidden states, our hidden states are defined as: ${{\textbf {z}}}_{t}=(z_{1t} \dotsc z_{St}\dot{z}_{1t} \dotsc \dot{z}_{St})$, where the first set of latent variables corresponding to $\varvec{\mu }_{t}$ and the second set of latent variables corresponding to $\varvec{\mu }_{t-1}$.

While the posterior effectively represents the detected symptom *s*, it still needs to be post-processed to obtain a binary symptom representation, so that it can be compared with ground truth data. We achieved this by using a simple thresholding by using *f*-measure calculations to find the optimum cut-off threshold points, $\theta _{s}$, for each symptom class and temporal state. To find the optimum cut-off point, we used Receiver Operating Characteristic (ROC) function and then computed $TPR + (1-FPR)$, where TPR is True Positive Rate and FPR is False Positive Rate. Then, we sorted the results and took average of top 5 optimum thresholds to be used in our testing phase.

## Evaluation

### Description of dataset

**Fig. 3:**
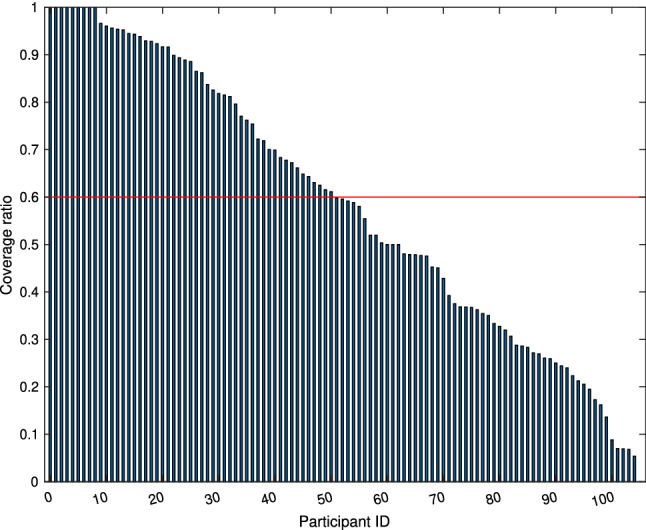
The ratio of personal exposure coverage. The red line indicates the 60% (50 participants) exposure coverage limit

Overall, 106 participants were recruited over a period of two years. Each was asked to carry the PAM and keep daily records for six months. We collected over 54 million data points. It is the largest data set of its kind in the world. Figure 3 shows the patient monitoring coverage during our study (up to 182 days). Reasons for low coverage included dropping out of the study, sensor malfunction, holiday periods and incapacitating illness.

**Fig. 4:**
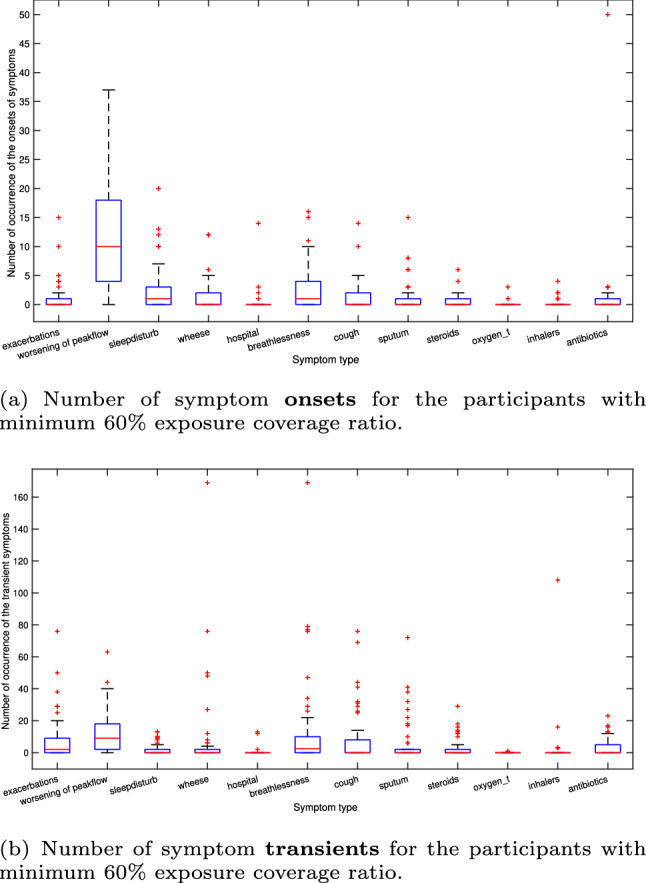
**a**, **b** show the number of occurrence of the symptom onsets and transients, respectively

Figure 4a depicts the number of symptom onsets and Fig. 4b depicts the number of symptom transients (i.e. healing period) for participants who carried our sensors for more than 60% of the time during our study ($n=50$). To examine the effect of exposure coverage, we conducted experiments with participants who carried our sensors for more than 60% of the observation as well as with no personal coverage threshold. The number of symptom onsets showed a large variance depending on the symptom. The highest variation was seen for worsening of peak flow measurements, on average 10 onsets. It is worth noting that only exacerbations and peak flow (lung capacity) were considered biomarkers, as others were based on the patients’ personal diary symptom records. ‘Exacerbations’ were assigned by a respiratory clinician upon completion based on a combination of symptoms, medication use and lung capacity. We calculated the median peak flow for each participant, then transformed the daily peak flow measurements into binary outputs based on the median (i.e. below the personal median 1 and above the personal median 0.). We named this biomarker, “worsening of peak flow”. We annotated the temporal states of the symptoms (i.e. onset, transient, offset) in order to use in our prediction models. The onset of a symptom was considered as the first day appearance of a symptom, transient of a symptom was considered as the recovery period between the first day appearance of a symptom and the last day appearance of a symptom, and offset was considered as the last day appearance of a symptom.

Most of the symptoms had a low number of onset occurrences, such as being hospitalised, using inhalers, steroids, experiencing sputum, taking oxygen. However, this was not the case for peak flow measurements where the number of occurrences and variance in worsening of peak flow measurements was high. It can be seen in Fig. 4b that there was frequent symptom transients in the cohort study. In other words, although the participants may not have experienced their symptoms very often, it could take a long time to recover. This posed a particular data analysis challenge; fewer symptom onsets and a data set dominated by transients.

**Table 1:** Results of Four-Way Analyses of Variance for the COPD symptom detection system

MANOVA I												
Source	Overall			Onset			Transient			Offset		
	*df*	*F*	$\eta ^{2}$	*df*	*F*	$\eta ^{2}$	*df*	*F*	$\eta ^{2}$	*df*	*F*	$\eta ^{2}$
CLSR	4	$$59.742{* * *}$$	0.262	4	$$614.663{***}$$	0.785	4	$$54.911{***}$$	0.246	4	$$566.991{***}$$	0.771
SYMP	11	$$1720.174{***}$$	0.738	11	$$133.965{***}$$	0.687	11	$$193.306{***}$$	0.760	11	$$102.574{***}$$	0.627
NOPA	1	$$3436.225{***}$$	0.836	1	$$898.448{***}$$	0.572	1	$$3555.974{***}$$	0.841	1	$$818.017{***}$$	0.549
SENS	2	1.162	0.003	2	2.267	0.007	2	$$6.758{**}$$	0.020	2	$$3.512{*}$$	0.010
CLSR$$\times$$SYMP	44	$$9.208{***}$$	0.376	44	$$19.177{***}$$	0.557	44	$$21.999{***}$$	0.590	44	$$15.056{***}$$	0.496
CLSR$$\times$$NOPA	2	$$130.117{***}$$	0.279	2	$$242.534{***}$$	0.419	2	$$115.257{***}$$	0.255	2	$$229.258{***}$$	0.406
CLSR$$\times$$SENS	8	$$8.310{***}$$	0.090	8	$$5.066{***}$$	0.057	8	$$8.546{***}$$	0.092	8	$$8.412{***}$$	0.091
SYMP$$\times$$NOPA	11	$$61.082{***}$$	0.500	11	$$11.472{***}$$	0.158	11	$$89.261{***}$$	0.594	11	$$9.506{***}$$	0.135
SYMP$\times$SENS	22	0.334	0.011	22	0.634	0.020	22	0.711	0.023	22	0.481	0.015
NOPA$$\times$$SENS	2	$$18.630{***}$$	0.053	2	$$12.453{***}$$	0.036	2	$$17.499{***}$$	0.050	2	$$22.933{***}$$	0.064
CLSR$$\times$$SYMP$$\times$$NOPA	22	$$14.719{***}$$	0.325	22	$$6.857{***}$$	0.183	22	$$48.011{***}$$	0.611	22	$$4.912{***}$$	0.139
CLSR$\times$SYMP$\times$SENS	88	0.564	0.069	88	0.518	0.064	88	0.479	0.059	88	0.675	0.081
CLSR$$\times$$NOPA$$\times$$SENS	4	$$5.798{***}$$	0.033	4	$$3.637{***}$$	0.021	4	$$6.003{***}$$	0.034	4	$$6.922{***}$$	0.040
SYMP$\times$NOPA$\times$SENS	22	0.601	0.019	22	0.275	0.009	22	1.082	0.034	22	1.299	0.041
CLSR$\times$SYMP$\times$NOPA$\times$SENS	44	0.647	0.041	44	0.498	0.032	44	0.687	0.043	44	0.901	0.056
Error	672			672			672			672		

$\eta ^2$ is the partial eta squared measure of effect size. The table demonstrates the statistical effect of main factors and the significant interactions between factors. Corresponding meanings of the abbreviations are as follows: *NOPA* number of participants, *CSLR* classifier, *SENS* sensor, *SYMP* symptoms

*$$p < 0.05$$ **$$p < 0.01$$

***$$p < 0.001$$

### Training and testing

Our experiments were designed to compute the F-measures for the combinations of the following factors:

- Classifiers (CLSR): RF, PLCA-3D, PLCA-LDS-3D, PLCA-4D, PLCA-LDS-4D.
- Symptoms (SYMP): Worsening of peak flow, exacerbations, sleep disturbance, wheese, breathlessness, cough, sputum, oxygen, inhalers, antibiotics.
- Number of participants (NOPA): personalised analysis, population analysis.
- Personal exposure coverage (PC): 60%, no threshold.
- Sensors (SENS): all sensors (i.e. NO, CO, PM1, PM 2.5, PM 10, Relative Humidity, triaxial accelerometer, temperature, audio from microphones, GPS data, and peak flow measurements); only air pollutants (i.e. NO, CO, PM1, PM2.5, PM10, relative humidity); only peak flow measurements.
- Spectral features (FEAT): only average; a rich set of spectral features (i.e. average, median, variance, maximum, minimum, spectral flux, kurtosis, entropy, energy, skewness).

### Understanding the effects of different factors

To test significance of the factors and their interaction, we conducted two four-way Multivariate Analyses of Variance (MANOVA) due to large numbers of subcategories. The first MANOVA (MANOVA-I) test involved number of participants (i.e. personalised versus population), classifiers (i.e. RF, PLCA-3D, PLCA-4D, PLCA-LDS-3D, PLCA-LDS-4D), sensors (i.e. all sensors including peak flow measurements, only air pollutants, only peak flow measurements), and symptoms (i.e. Worsening of peak flow, worsening of exacerbations, sleep disturbance, wheese, breathlessness, cough, sputum, oxygen, inhalers, antibiotics) as independent variables. The second MANOVA (MANOVA-II) test involved classifiers, symptoms, personal coverage threshold (i.e. minimum of 60% and no coverage limit), and features (i.e. rich spectral feature set, only average) as independent variables. There were four independent variables in both of the MANOVA tests: the F1-measure values for the overall symptom detection, F1-measure values for temporal state detection, namely, onset, transient, and offset. The statistical MANOVA-I results are presented in Table 1 and MANOVA-II results are presented in Table 2. The definitions in [23] have been adopted to discuss the effect sizes: small effect size ($\eta ^2 \le 0.01$), medium effect size ($0.01 \le \eta ^2 \le 0.06$) and large effect size ($0.06 \le \eta ^2 \le 0.14$). We also provided supplementary materials for the 95% confidence interval differences between classifiers in Appendix A.1 and symptoms in Appendix A.2, and additional visualisations of results in Appendix A.3.

#### Classifiers

There was a highly significant effect of the classifiers (*CLSR*) on the overall and temporal symptom states predictions. The overall symptom prediction results were highly significant with $F(4, 672)=59.742, p<.001$. The effect size of the classifier factor was very large, $\eta ^2=.262$. Similarly, the prediction of all temporal symptom states were highly significant with very large effect sizes: $F(4, 672)=614.663, p<.001$, $\eta ^2=.785$ for Onsets; $F(4, 672)=54.911, p<.001$, $\eta ^2=.246$ for Transients; $F(4, 672)=566,991, p<.001$, $\eta ^2=.771$ for Offsets.

**Fig. 5:**
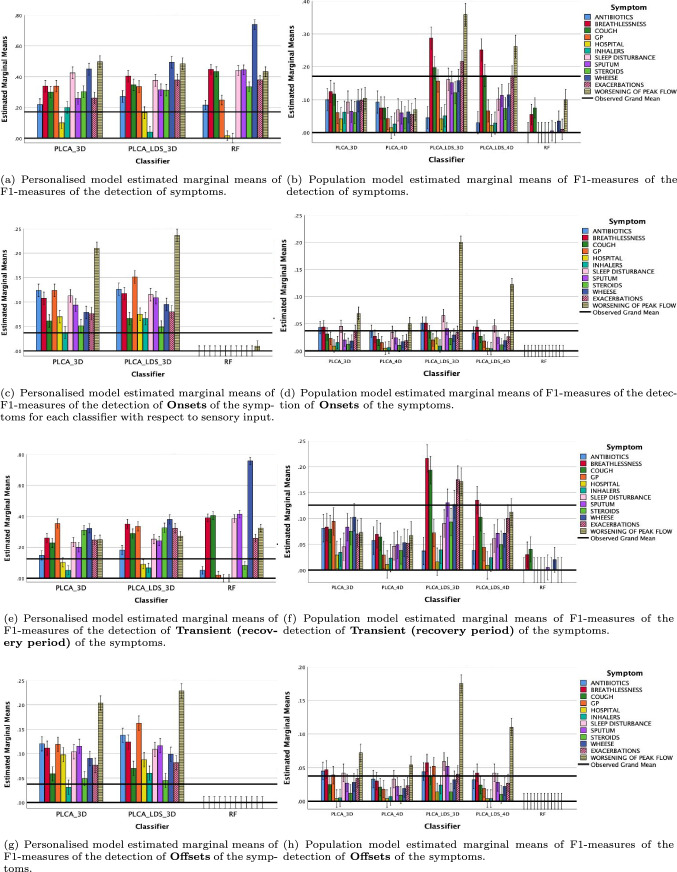
The F1-measure values of each classifier obtained in the detection of symptoms and their temporal states, namely, onset, transient, and offset. **a**, **b** show the symptom detection results. **c**–**h** show the detection of temporal states of symptoms. The error bars depict the 95% confidence interval. The results are normalised between 0 and 1. The F1 measures presented for three highly significant factors found in the MANOVA test, namely, classifier, number of participants (i.e. personalised versus population), and symptoms

The posthoc analyses (multiple comparison procedure, Bonferroni) showed that for the overall symptom predictions, the PLCA-LDS-3D predictions ($\mu =0.201$) were significantly higher than the rest of the classifiers ($p<0.001$). Similarly, the PLCA-LDS-3D performed better than the rest of the models in the detection of temporal states of the symptoms. Figure 5a, b shows the overall results obtained for the detection of the symptoms for each classifier in personalised and population categories. For the personalised predictions, the highest average result was obtained by optimised RF model ($\mu$ = 0.34, $\sigma$ = 0.19); however, overall results were competitive. The PLCA LDS 3D ($\mu$ = 0.33, $\sigma$ =0.13) and PLCA 3D ($\mu$ = 0.31, $\sigma$ = 0.12) models obtained very close results to the RF model with smaller standard deviations. Therefore, it is possible to argue that the PLCA LDS 3D and PLCA 3D classifiers are reasonably more robust models compared to the RF model. In the population predictions, the PLCA LDS 3D model produced the best results by far ($\mu$ = 0.17, $\sigma$ = 0.13), whereas PLCA 3D ($\mu$ = 0.11, $\sigma$ = 0.12) and PLCA LDS 4D ($\mu$ = 0.12, $\sigma$ = 0.10) performed reasonably well. On the contrary, the worst results were obtained by PLCA 4D ($\mu$ = 0.06, $\sigma$ = 0.03) and RF models ($\mu$ = 0.02, $\sigma$ = 0.04).

Figure 5c–h shows the overall results obtained for the detection of the temporal states of symptoms, namely, onset, transient, and offset. For the personalised predictions, the PLCA-LDS 3D model gave the best results for all temporal states (Onset: $\mu =11$%, $\sigma =5$%; Transient: $\mu =26$%, $\sigma =10$%; Offset: $\mu =11$%, $\sigma =5$%), whereas the RF model only performed well in the detection of temporal states (onset: $\mu =0$%, $\sigma =0$%; transient: $\mu =26$%, $\sigma =23$%; offset: $\mu =0$%, $\sigma =0$%). For the population predictions, although the PLCA LDS 3D model gave the best results (onset: $\mu =6$%, $\sigma =6$%; transient: $\mu =13$%, $\sigma =10$%; offset $\mu =5$%, $\sigma =5$%), overall results were fairly low. The RF model did not perform well on the population analysis (onset: $\mu =0$%, $\sigma =0$%; transient $\mu =1$%, $\sigma =1$%; offset: $\mu =0$%, $\sigma =0$%). The rest of the classifiers did not perform as well as PLCA-LDS 3D model. The average performance, $\Delta \mu$, in the detection of temporal states for the PLCA 4D and PLCA LDS 4D were between 0.02 and 0.06%. Overall the average optimum parameters discovered by using Randomised Search algorithm for the RF model were as follows: number of estimators = 155.2, min. samples split = 5.66, min. samples leaf = 5.77, max. depth = 54.4.

The performance of the PLCA LDS 3D can be explained by the fact that it takes into account the transition of symptoms, and therefore, it performs better in capturing the temporal aspects of the symptoms whilst the PLCA and RF models do not incorporate such computation. The results obtained for the PLCA 4D and PLCA-LDS-4D were relatively lower than the PLCA-LDS-3D and PLCA 3D models. One reason could be that we may need more data to capture the seasonal effect of environmental measurements on patients’ symptoms. Another explanation could be that the traditional way of splitting a year into seasons by three months may not reflect the actual seasonal variations in the UK. It can also be difficult to measure the seasonal effect when patients are not very active and do not frequently go out.

#### Symptoms

There was a highly significant effect of the type of symptoms (*SYMP*) on the prediction of symptoms $F(11, 672)=172.174, p<0.001$) and their temporal states, Onset: $F(11, 672)=133.965, p<0.001$, Transient: $F(11, 672)=193.306, p<0.001$), Offset: $F(11, 672)=102.574, p<0.001$). The effect size for the detection of symptoms ($\eta ^2=0.738$) and their temporal states (onset: $\eta ^2=0.687$, transient: $\eta ^2=0.760$, offset: $\eta ^2=0.627$) were very large. The posthoc analyses showed that there was significant difference between worsening of peak flows and other symptoms ($p<0.001$) except for wheeze. For the onset and offset detection of the symptoms, the best results were obtained for the worsening of peak flow. The average differences between the detection of worsening of peak flow and all other symptoms were highly significant ($p<0.001$). On the contrary, for the transient detection, the best results were obtained for wheeze. The average differences between the detection of wheeze and all other symptoms were highly significant ($p<0.001$).

It is evident that high frequency prevalence of wheeze symptoms in the personalised analysis increased the overall performance in the detection of this symptom. This is more likely to be caused by the filtering strategy used in the training and testing phase in the personalised analysis. Additionally, there were reasonable results in the detection of the biomarkers, namely, exacerbations and worsening of peak flow. The overall average and standard deviation of results in the detection of exacerbation was $\mu = 19$% with $\sigma = 1$% and in the detection of worsening of peak flow was $\mu = 27.7$% with $\sigma = 2$%.

**Table 2:** Results of Four-Way Analyses of Variance for the COPD symptom detection system

MANOVA II												
Source	Overall			Onset			Transient			Offset		
	df	*F*	$\eta ^{2}$	df	*F*	$\eta ^{2}$	df	*F*	$\eta ^{2}$	df	*F*	$\eta ^{2}$
CLSR	4	$$21.298{***}$$	0.106	4	$$155.201{***}$$	0.463	4	$$20.537{***}$$	0.102	4	$$163.331{***}$$	0.476
SYMP	11	$$10.305{***}$$	0.136	11	$$29.421{***}$$	0.310	11	$$9.359{***}$$	0.125	11	$$21.680{***}$$	0.249
PC	1	0.181	0.00	1	3.458	0.005	1	0.073	0.00	1	0.442	0.001
FEAT	1	0.734	0.001	1	0.931	0.001	1	0.058	0.00	1	0.047	0.00
CLSR$$\times$$SYMP	44	1.336	0.075	44	$$4.834{***}$$	0.228	44	$$2.497{***}$$	0.132	44	$$3.755{***}$$	0.187
CLSR$\times$PC	4	0.266	0.001	4	0.481	0.003	4	0.174	0.001	4	1.170	0.006
CLSR$\times$FEAT	4	0.619	0.003	4	0.833	0.005	4	0.171	0.001	4	2.111	0.012
SYMP$$\times$$PC	11	0.682	0.010	11	$$3.232{***}$$	0.047	11	0.470	0.007	11	$$2.861{**}$$	0.042
SYMP$\times$FEAT	11	0.209	0.003	11	0.876	0.013	11	0.147	0.002	11	0.632	0.010
PC$\times$FEAT	1	0.400	0.001	1	0.232	0.00	1	0.040	0.00	1	0.011	0.00
CLSR$\times$SYMP$\times$PC	44	0.266	0.016	44	1.145	0.065	44	0.289	0.017	44	0.727	0.043
CLSR$\times$SYMP$\times$FEAT	44	0.278	0.017	44	0.562	0.033	44	0.117	0.007	44	0.502	0.030
CLSR$\times$PC$\times$FEAT	4	0.933	0.005	4	0.228	0.001	4	0.138	0.001	4	1.503	0.008
SYMP$\times$PC$\times$FEAT	11	0.360	0.005	11	0.354	0.005	11	0.296	0.005	11	0.186	0.003
CLSR$\times$SYMP$\times$PC$\times$FEAT	44	0.343	0.021	44	0.481	0.029	44	0.209	0.013	44	0.331	0.020
Error	720			720			720			720		

$\eta ^2$ is the partial eta squared measure of effect size. The table demonstrates the statistical effect of main factors and the significant interactions between factors. Corresponding meanings of the abbreviations are as follows: *CSLR* classifier, *FEAT* feature set, *SYMP* symptoms, *PC* personal coverage

$$*p < 0.05$$

$${**}p < 0.01$$

$${***}p < 0.001$$

#### Influence of number of participants

The analysis of variance showed that the effect of number of participants (*NOPA*) was highly significant for the detection of symptoms $F(1,672)=3436.225, p<.001$ and their temporal states, Onset: $F(1,672)=898.448, p<.001$, Transient: $F(1,672)=3555.974, p<.001$, Offset: $F(1,672)=818.017, p<.001$. The effect sizes were found to be very large in the detection symptoms ($\eta ^2=0.836$) and their temporal states (Onset: $\eta ^2=0.785$, Transient: $\eta ^2=0.841$, Offset: $\eta ^2=0.549$).

The overall symptom detection results obtained from personalised models were higher than the population model (PERS: $\mu =$ 32%and $\sigma =$ 16%, POPU: $\mu =$ 10% and $\sigma =$ 11%). There was a similar pattern in the detection of the temporal states of symptoms. The performances in the detection of the temporal states for the personalised models were $\mu =$ 6% with $\sigma =$ 6% for the detection of onsets, $\mu =$ 25% with $\sigma =$ 17% for the detection of transient, $\mu =$ 6% with $\sigma =$ 6% for the detection of offsets. On the other hand, there were lower results for the population models. The performances in the detection of the temporal states for the population models were $\mu =$ 3% with $\sigma =$ 3% for the detection of onsets, $\mu =$ 6% with $\sigma =$ 6% for the detection of transient, $\mu =$ 3% with $\sigma =$ 3% for the detection of offsets. While the results indicate that the overall performance of the personalised models in the detection of symptoms and their temporal states were clearly better than the population models, it is worth to point out that there were fewer symptoms used in the personalised models since we only used the symptoms occurred with all three temporal states both in the training (i.e. first half of each month) and testing phase (i.e. second half of each month) for each participant. Therefore, the personalised models are more likely be overtrained where as the population models are more likely to be undertrained.

#### Sensory input

Our results showed that there was no significant effect of the choice of sensory input (*SENS*) in the detection of overall symptoms. There was significant effect of the choice of sensory input on the detection of transient state of the symptoms $F(1,672)=6.758, p<.05$ with a medium effect size, $\eta ^2=0.020$, and there was significant effect on the detection of the offsets of the symptoms, $F(1,672)=3.512, p<.05$ with a small effect size, $\eta ^2=0.010$.

There was a very small difference between different types of sensory inputs for the detection of symptoms and their temporal states ($-1\% \le \Delta \le 1\%$). The results indicate that the quality of an individual’s environment, specifically air pollutants, are as good predictors of the worsening of respiratory symptoms in COPD patients as a direct measure of changes in their acute lung capacity. It was interesting to see that when we used only peak flow measurements as input, the results for the detection of worsening of peak flow measurement was still not very high. This can be explained by the fact that there was significant variation in the number of onsets of the worsening of peak flows (see Fig. 4a). It could be inferred therefore that the low performance results may more likely to be caused by worsening of peak flow measurements with short transients. The results show that using only peak flow measurements or only air pollution measurements are also sufficient for the prediction of symptoms.

#### Personal coverage

There was no significant effect of the personal coverage (*PC*) in our predictions even though the results obtained in the detection of onsets were the closest to show a significant effect, $p=.06$. The average F1 measurements for the 60% personal coverage threshold was $\mu =$ 17% with $\sigma =$ 16% and for no threshold was $\mu =$ 18% with $\sigma =$17%. The performance in the detection of the temporal states for the 60% personal coverage threshold were $\mu =$ 4% with $\sigma =$ 4% for the detection of onsets, $\mu =$ 13% with $\sigma =$ 13% for the detection of transients, $\mu =$ 4% with $\sigma =$ 5% for the detection of offsets. In parallel, there were similar results when we did not use any threshold in the models. The performance in the detection of the temporal states for no threshold models were $\mu =$ 3% with $\sigma =$ 3% for the detection of onsets, $\mu =$ 13% with $\sigma =$ 3% for the detection of transients, $\mu =$ 3% with $\sigma =$ 3% for the detection of offsets. This implies that using only indoor monitoring measurements may be sufficient to cover some of the COPD patients’ symptoms as they may not be very active in their daily lives. However this outcome requires further research since personal air pollution exposure and its health effects are a relatively new research field and may involve many complex elements.

#### Feature sets

One of the important contributions of our study was that we used a rich set of features (*FEAT*) in our analysis to better capture the cumulative, acute and magnitude-related symptoms. However, when we compared the rich set of feature sets against only computing average for each sliding window, we found that there was no significant effect of the choice of feature sets in our study. The overall results were very similar to each other (rich feature set: $\mu =$ 17%,$\sigma =$ 16%; only average: $\mu =$ 16%, $\sigma =$ 17%). Similarly, there was similar pattern in the detection of temporal states, where the results varied between $\mu =$ 3% and $\sigma =$ 5% for the detection of onset and offset of the symptoms for both of the feature sets, and $\mu =$ 12% and $\sigma =$ 13% for the detection of transients.

#### Interaction between the factors

There was highly significant interactions between *CLSR*, *SYMP*, and *NOPA*. All of the interactions between these three factors were highly significant with very large effect sizes. Although there was no significant effect of *SENS* factor on the detection of symptoms, there was highly significant effect of *SENS* factor on the detection of transients $F(2, 672)=6.758, p<.01$ with medium effect size $\eta ^2=0.020$, and there was significant effect on the detection of offsets, $F(2, 672)=3.512, p<.01$, with small effect size $\eta ^2=0.010$. When *SENS* interacted with *CLSR*, there was a highly significant effect with medium effect size for the detection of symptoms, $F(8, 672)=8.310, p<.001$, $\eta ^2=0.090$, and for the temporal symptom states: Onset: $F(8, 672)=5.066, p<.001$, $\eta ^2=0.057$, Transient: $F(8, 672)=8.546, p<.001$, $\eta ^2=0.092$, Offset: $F(8, 672)=8.412, p<.001$, $\eta ^2=0.091$. Moreover, there was highly significant effect with a medium effect size on the detection of symptoms when *SENS* interacted with *CLSR* and *NOPA*, $F(4, 776)=5.798, p<.001$, $\eta ^2=0.033$. There was highly significant effect with a medium effect size on the detection of temporal symptom states: Onset: $F(4, 672)=3.637, p<.001$, $\eta ^2=0.021$, Transient: $F(4, 672)=6.003, p<.001$, $\eta ^2=0.034$, Offset: $F(4, 672)=6.922, p<.001$, $\eta ^2=0.040$.

### Discussion

Overall, our results were not very high. However, unlike the forecasting model applied to questionnaire data in [7], our prediction models continued to make a prediction even during the transient of symptoms (i.e. recovery period) instead of limiting the symptoms only to onsets. While removing the recovery period of patients from the data set could significantly improve the training and testing of the model, it could cause overfitting, since there would be a very small amount of available data. We believe that our study would be the closest application to a real-world scenario. It is evident that there is a small variation in all our model runs, depending on the type of input and features used. Despite the moderate performance of models in forecasting outcomes, it is possible to observe that PLCA-LDS model considerably outperformed Random Forest. The results obtained for the PLCA-LDS-4D was lower than the PLCA-LDS-3D model. One reason could be that more data are needed to capture the seasonal effect of environmental measurements on patients’ symptoms. It can also be difficult to measure seasonal effects when patients are not very active and do not frequently go out. Additionally, this can partly be explained by large numbers of missing data in the seasonality dimension, *m*, of the four-dimensional tensor dictionary and the method used in the imputation process. While the participants took part in the study for “a maximum of six months”, we utilised a traditional two-dimensional approach (i.e. $p \times (m \times s \times a)$) in the imputation process. Therefore, it is possible that the large numbers of missing data, as well as the utilisation of two-dimensional imputation process, might have shown a negative effect on the results of the PLCA-4D and PLCA-LDS 4D models. Moreover, it would be beneficial to investigate using random accelerations and determine its impact on the PLCA-LDS models.

Our study is the largest of its kind anywhere in the world and the first study to investigate the effects of environmental factors on COPD patients’ daily symptoms and forecast the symptoms one day in advance. By utilising environmental factors in the prediction, we aimed to design behavioural interventions to reduce risk of worsening of symptoms and/or exacerbation. These interventions would be in addition to any clinical interventions. It can help predict what environmental conditions cause a worsening of symptoms for an individual, can assist clinicians to give personalised advice in rehabilitation as to how COPD patients can change their behaviour and living conditions to improve health and quality of life.

## Conclusions

In this study, we presented our results on the prediction of the worsening of COPD patients’ daily symptoms one day in advance by using sensory observations. In general, reasonable results were obtained for all of the classifiers. Although the predictions were not always accurate, the PLCA-LDS 3D model outperformed other PLCA models and RF model. We found that there was significant difference between the classifiers, symptoms and the personalised versus population analysis. We have also shown that indicators of the quality of an individual’s environment, specifically air pollutants, are as good predictors of the worsening of respiratory symptoms in COPD patients as a direct measure of changes in their acute lung capacity. It should be noted that it is difficult to monitor personal exposure in a free living cohort, such as ours; we had no control over their behaviour and patients’ frequently failed to carry the sensory devices when they left their home. Nonetheless, there was no significant effect found for personal coverage threshold in our experiments.

There may also be some other possible factors affecting our model results. We are aware of the fact that the personalised predictions are highly likely to be affected by over-fitting, since they are trained with a relatively small data set per participant, compared to other modelling scenarios. Conversely, it is possible that when we conducted our population experiments on the far larger data set, the opposite effect may have occurred, i.e. they might be under-trained. This may explain why we found significant difference between personalised and population experiments. There was also significant effect of the choice of classifier and the type of symptoms in our experimental results.

## Figures and Tables

**Fig. 6:**
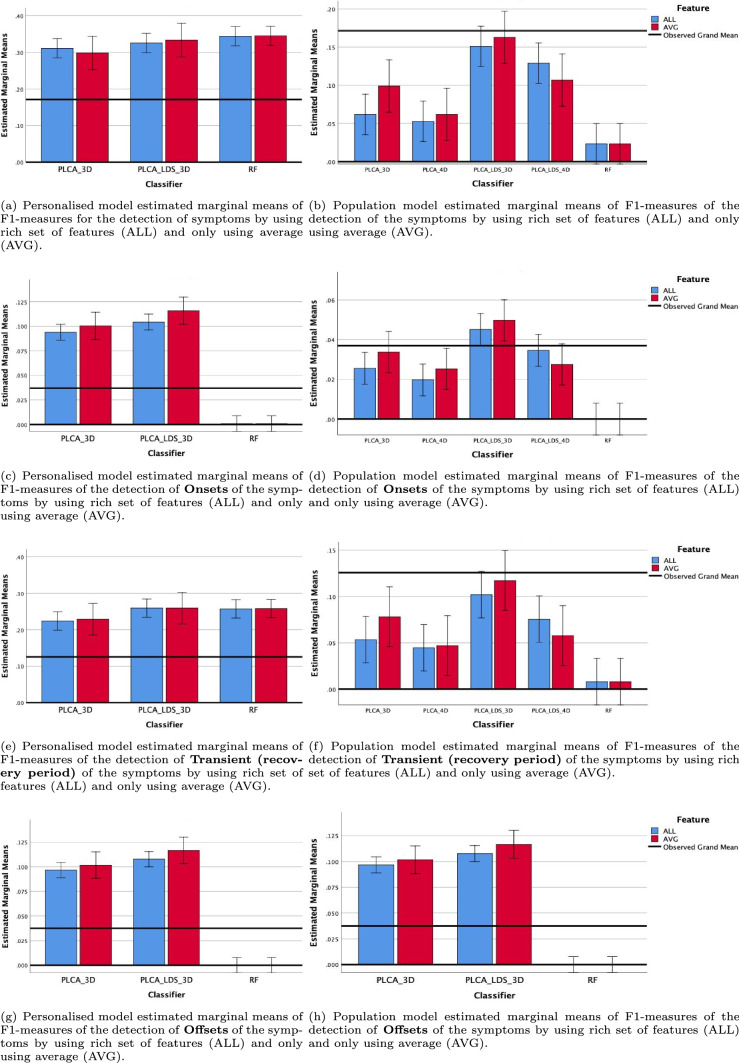
The figures show the marginal means of F1-measures of the prediction of the symptoms and their temporal states for the rich set of features and only using average in the predictions of personalised and population models. The error bars depict the 95% confidence interval. The horizontal black lines indicates the observed grand average

**Fig. 7:**
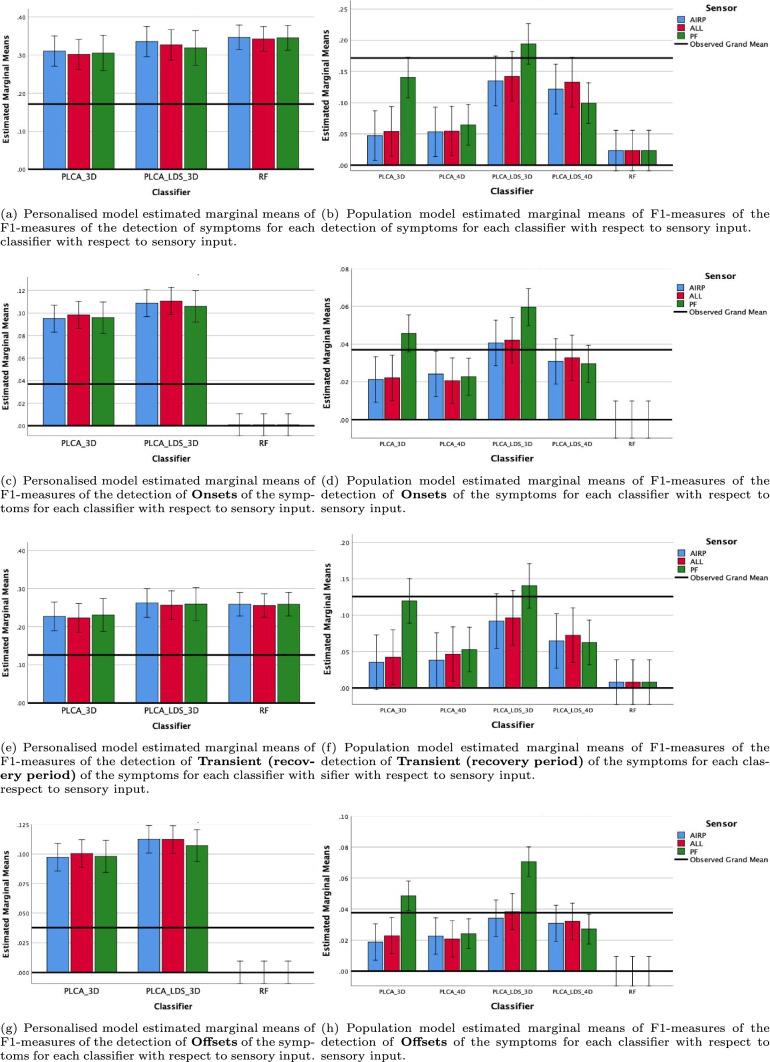
The figures show the marginal means of F1-measures of the prediction of the symptoms and their temporal states for each classifier with respect to the sensory input of all sensors (ALL), only air pollutants (AIRP), and only peak flow measurements (PF) in the predictions of personalised and population models. The error bars depict the 95% confidence interval. The horizontal black lines indicates the observed grand average

**Fig. 8:**
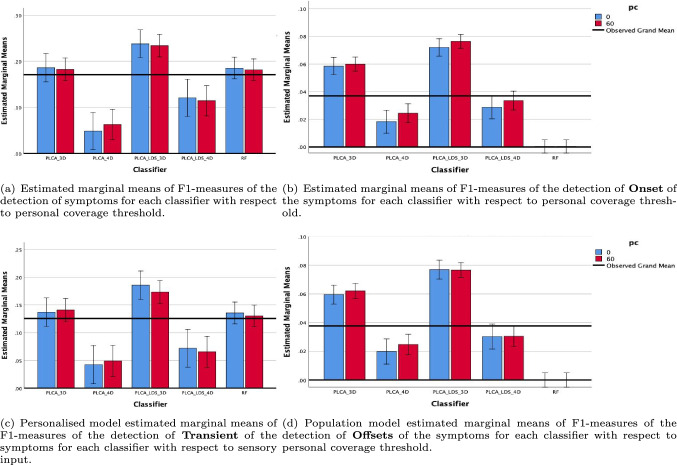
The figures show the marginal means of F1-measures of the prediction performance of each classifier with respect to the personal coverage thresholds (pc): 60% and 0% threshold (i.e. no threshold). The error bars depict the 95% confidence interval. The horizontal black lines indicates the observed grand average

## Data Availability

The PLCA code: https://github.com/skolozali/cope_study. Data will be made publicly available on the IEEE Data Port upon publication of the manuscript.

## Appendix A.1: The 95% confidence interval differences for the classifier and symptom factors

We provide the 95% confidence interval differences for the classifier and symptom factors in this section.

### A.1 Classifier

For the overall symptom predictions, the PLCA-LDS-3D predictions ($\mu =$ 0.201) were significantly higher than the rest of the classifiers ($p<$ 0.001). The average F-measure 95% confidence interval differences between the PLCA-LDS-3D classifier and the rest of the classifiers were as follows: the RF ($4\% \le \Delta \le 7\%$), PLCA-3D ($4\% \le \Delta \le 7\%$), PLCA-4D ($16\% \le \Delta \le 20\%$), and PLCA-LDS-4D models ($10\% \le \Delta \le 13\%$).

Similarly, the PLCA-LDS-3D performed better than the rest of the models in the detection of temporal states of the symptoms. The average F-measure 95% confidence interval differences between the PLCA-LDS-3D classifier and the RF model were between $7\% \le \Delta \le 8\%$ for the onset detection, were between $4\% \le \Delta \le 6\%$ for the transient detection, and were between $7\% \le \Delta \le 8\%$ for the offset detection. The difference was smaller between the PLCA-LDS-3D and the other classifiers. The 95% confidence interval differences of PLCA-LDS-3D compared to the rest of the classifiers were as follows: PLCA-3D (Onset: $1\% \le \Delta \le 1\%$, Transient: $3\% \le \Delta \le 5\%$, Offset: $1\% \le \Delta \le 2\%$), PLCA-4D (Onset: $5\% \le \Delta \le 6\%$, Transient: $12\% \le \Delta \le 15\%$, Offset: $5\% \le \Delta \le 6\%$), and PLCA-LDS-4D models (Onset: $4\% \le \Delta \le 5\%$, Transient: $10\% \le \Delta \le 12\%$, Offset: $4\% \le \Delta \le 5\%$).

### A.2: Symptoms

The best results were obtained for the worsening of peak flow. The 95% confidence interval on the difference of the average F1 measures between worsening of peak flow and other symptoms were as follows: antibiotics ($13\% \le \Delta \le 19\%$), breathlessness ($1\% \le \Delta \le 6\%$), cough ($3\% \le \Delta \le 10\%$), visiting GP ($10\% \le \Delta \le 17\%$), hospitalised ($20\% \le \Delta \le 27\%$), inhalers ($20\% \le \Delta \le 27\%$), sleep disturbance ($4\% \le \Delta \le 11\%$), sputum ($7\% \le \Delta \le 14\%$), steroids ($10\% \le \Delta \le 17\%$), exacerbations ($5\% \le \Delta \le 12\%$), and wheeze ($-1\% \le \Delta \le 5\%$).The average differences between the detection of worsening of peak flow and other symptoms were highly significant too ($p<$ 0.001) except for wheeze.

For the onset detection of the symptoms, the best results were obtained for the worsening of peak flow. The average difference of the F1 values between worsening of peak flows and other symptoms were as follows: antibiotics ($5\% \le \Delta \le 6\%$), breathlessness ($5\% \le \Delta \le 7\%$), cough ($6\% \le \Delta \le 8\%$), visiting GP ($5\% \le \Delta \le 7\%$), hospitalised ($7\% \le \Delta \le 9\%$), inhalers ($8\% \le \Delta \le 10\%$), sleep disturbance ($5\% \le \Delta \le 7\%$), sputum ($6\% \le \Delta \le 8\%$), steroids ($7\% \le \Delta \le 9\%$), exacerbations ($6\% \le \Delta \le 8\%$), and wheeze ($6\% \le \Delta \le 8\%$).The average differences between the detection of worsening of peak flow and all other symptoms were highly significant as well ($p<$ 0.001).

On the contrary, for the transient detection, the best results were obtained for wheeze. The average F1 values between worsening of peak flow and other symptoms were as follows: antibiotics ($14\% \le \Delta \le 19\%$), breathlessness ($2\% \le \Delta \le 6\%$), cough ($4\% \le \Delta \le 8\%$), visiting GP ($11\% \le \Delta \le 15\%$), hospitalised ($18\% \le \Delta \le 23\%$), inhalers ($18\% \le \Delta \le 22\%$), worsening of peak flow ($6\% \le \Delta \le 10\%$), sleep disturbance ($7\% \le \Delta \le 11\%$), sputum ($6\% \le \Delta \le 10\%$), steroids ($10\% \le \Delta \le 14\%$), and exacerbations ($6\% \le \Delta \le 10\%$). The average differences between the detection of wheeze and all other symptoms were highly significant ($p<$ 0.001).

The offset detection results were very similar to the onset detection results. The best results were again obtained for the offset detection of the worsening of peak flow measurements. Similar to the onset detection results, the overall difference between the worsening of peak flow measurements and the rest of the symptom prediction results were between $4\% \le \Delta \le 9\%$. The average F1 value difference between worsening of peak flows and other symptoms were as follows: antibiotics ($4\% \le \Delta \le 6\%$), breathlessness ($4\% \le \Delta \le 6\%$), cough ($6\% \le \Delta \le 8\%$), visiting GP ($4\% \le \Delta \le 6\%$), hospitalised ($6\% \le \Delta \le 8\%$), inhalers ($7\% \le \Delta \le 9\%$), sleep disturbance ($4\% \le \Delta \le 6\%$), sputum ($5\% \le \Delta \le 6\%$), steroids ($7\% \le \Delta \le 9\%$), exacerbations ($5\% \le \Delta \le 7\%$), and wheese ($5\% \le \Delta \le 7\%$). The average differences between the detection of worsening of peak flow and all other symptoms were highly significant ($p<$ 0.001).

### A.3 Visual representation of the results obtained with different factors

In this section, we provide the summarised results obtained for the interaction between classifiers and features in Fig. 6, classifiers and sensors in Fig. 7, and classifiers and personal coverage thresholds in Fig. 8. These results were the outcome of our statistical analysis used to investigate the effect of different factors (see Sect. 4.3).

## Footnotes

1 https://archive.ics.uci.edu/ml/index.php.
